# Raloxifene/SBE-β-CD Inclusion Complexes Formulated into Nanoparticles with Chitosan to Overcome the Absorption Barrier for Bioavailability Enhancement

**DOI:** 10.3390/pharmaceutics10030076

**Published:** 2018-06-28

**Authors:** Zaihua Wang, Yan Li

**Affiliations:** 1 Guangzhou Guocaoxiafang Biotechnology Co. Ltd., No. 3 Luoxuan 4th Road, Guangzhou International Biotech Island, Guangzhou 510320, China; 2 Department of Pharmacy, School of Pharmacy, No. 601 West Huangpu Avenue, Jinan University, Guangzhou 510632, China; pharmazxw@yeah.net

**Keywords:** raloxifene, bioavailability, nanoparticles, cyclodextrin, chitosan, electrostatic complexation

## Abstract

Raloxifene (RXF) is a hormone-like medication used for treating postmenopausal osteoporosis and estrogen-dependent breast cancer, yet associated with bad low bioavailability due to poor solubility. This study was intended to develop cyclodextrin/chitosan nanoparticles (ccNPs) for oral delivery of RXF in order to enhance the oral bioavailability. RXF-loaded ccNPs (RXF-ccNPs) were prepared by cyclodextrin inclusion followed by complexation with chitosan. RXF-ccNPs were fully characterized by particle size, morphology and in vitro drug release. The oral delivery efficacy and transepithelial transport potential were evaluated by pharmacokinetics, in situ single-pass intestinal perfusion, cellular uptake and ex vivo imaging. The resulting RXF-ccNPs were around 165 nm in particle size with a narrow distribution. The oral bioavailability of RXF was enhanced by 2.6 folds through ccNPs compared to RXF suspensions in rats. It was shown that RXF-ccNPs could improve the intestinal permeability of RXF, increase the cellular uptake of RXF and facilitate its transport across the absorptive epithelia. The results indicate that our developed ccNPs based on sulfobutylether-β-cyclodextrin and oligochitosan are a promising vehicle to orally deliver poorly water-soluble drugs over and above RXF.

## 1. Introduction

Raloxifene (RXF) is clinically used as a selective estrogen receptor modulator for the treatment of postmenopausal osteoporosis and estrogen-dependent breast cancer [1,2]. The absolute bioavailability RXF has been reported to be merely 2% following oral administration [3,4]. The major reasons lie in its extremely poor solubility and significant first-pass effect in the intestinal tract. RXF is not only insoluble in water, but also poorly soluble in oils and lipid excipients, showing an amphiphobic property. The amphiphobic property makes it hard to be formulated into suitable dosage forms other than conventional tablets and capsules. Intravenous injection of RXF seems to be impractical due to poor water-solubility. Therefore, we were inspired to develop advanced oral formulation to address the bioavailability issue.

Poor bioavailability tends to result in an unsatisfactory therapeutic effect, especially in the treatment of breast cancer. In order to break through the bottleneck of absorption, a variety of formulation strategies have been explored for oral delivery of RXF, including solid dispersions [5], lipid nanoparticles [6], nanoemulsions [7], mesoporous carbon nanospheres [8], nanomicelles [9], and polymeric nanoparticles [4,10]. However, these systems provisionally solve the entrapment of RXF, but ignore the serious drug expulsion and leakage upon standing storage. In most of nanocarriers, RXF would precipitate from the nanoparticles as subjected to centrifugation or standing. In order to enhance the long-term stability, Burra et al. [11] conducted a lyophilization process upon RXF-loaded solid lipid nanoparticles. Of note, the accomplishment of encapsulation is far from enough for oral delivery of RXF, an eligible delivery system should be provided with excellent transepithelial transport capacity. As known, cyclodextrins have the potential to effectively solubilize poorly water-soluble drugs [12], and chitosans have the advantage to overcome the absorptive barrier by loosening the tight junctions of enterocytes [13]. Nevertheless, these two functional materials are rarely used together for oral drug delivery.

In this study, we utilized anionic sulfobutylether-β-cyclodextrin (SBE-β-CD) and cationic oligochitosan to fabricate composite nanoparticles in an attempt to achieve RXF loading and bioavailability enhancement. RXF-loaded cyclodextrin/chitosan nanoparticles (RXF-ccNPs) were prepared via inclusion/electrostatic complexation and characterized with particle size, morphology, in vitro release and gastrointestinal stability. The oral delivery efficacy of nanoparticles for RXF was evaluated by in situ single-pass intestinal perfusion, cellular uptake, and oral pharmacokinetics.

## 2. Materials and Methods

### 2.1. Materials

Raloxifene hydrochloride (RXF) was purchased from Guangzhou Yiqing Biotechnology Co., Ltd. (Guangzhou, China). Sulfobutylether-β-cyclodextrin (SBE-β-CD) and water-soluble oligochitosan (MW < 2000) were obtained from Meilun Biotechnology Co., Ltd. (Dalian, China). CY3-labeled chitosan (CY3-chitosan) was from Ruixi Biological Technology Co., Ltd. (Xi’an, China). HPLC-grade methanol was provided by Merck (Darmstadt, Germany). Deionized water was manufactured by a water purifying system (Woter, Chengdu, China). All other chemicals or reagents were of analytical grade.

### 2.2. Phase Solubility Study

Phase solubility diagram of RXF as a function of SBE-β-CD was plotted according to the reported procedure [14]. Briefly, a slightly excess of RXF was added into 5 mL of distilled water, in which different concentrations of SBE-β-CD were previously preset (0, 10, 20, 40, 60, 80, 100 and 120 mg/mL). The mixtures were stirred with a magnetic stirrer for 48 h in a sealed bottle at 1000 rpm and 25 °C. Then, the samples were centrifuged using a centrifuge at 10,000 g for 5 min. RXF concentration in the supernatant was analyzed by high-performance liquid chromatography (HPLC) established below. The phase solubility diagram was drawn with the solubility of RXF to increased SBE-β-CD concentration. The complex formation constant (*K*_f_) was calculated based on the equation: *K*_f_ = *S*/*S*_0_(1 − *S*), where *S* and *S*_0_ denote the slope of linear equation (if applicable) and drug solubility in the absence of SBE-β-CD, respectively.

### 2.3. Preparation of Raloxifene -Loaded Cyclodextrin/Chitosan Nanoparticles (RXF-ccNPs)

RXF-ccNPs were prepared through two steps. The first step was to prepare RXF/SBE-β-CD inclusion complexes (RXF-SICs) by the solvent evaporation method [15]. In the second step, the resulting RXF-SICs were electrostatically conjugated with chitosan to form RXF-ccNPs. Briefly, RXF and SBE-β-CD were dissolved in 75% ethanol (*v*/*v*) and then evaporated to dry under reduced pressure at 35 °C. The residues were subsequently dissolved with an appropriate amount of deionized water to result in a solution equivalent to 10 mg/mL of SBE-β-CD. Finally, the obtained RXF-SICs solution was stepwise dropped into a 5 mg/mL of chitosan solution that was previously prepared with oligochitosan in water. To gain a preferred formulation, particle size and polydispersity (PDI) of RXF-ccNPs as indices were adopted to optimize the mass ratio of SBE-β-CD to oligochitosan upon complexation.

### 2.4. Characterization of RXF-ccNPs

The particle size and ζ potential of RXF-ccNPs were measured at 25 °C by Zetasizer Nano ZS (Malvern, Worcestershire, UK). The sample was properly diluted with deionized water and then subjected to laser diffraction or Doppler velocimetry. The data were collected with the built-in software for output of particle size ζ potential. The morphology of RXF-ccNPs was inspected by transmission electron microscopy (TEM, Tecnai series, Philips, Amsterdam, The Netherlands). The sample was diluted and fixed on a carbon-coated copper grid by drying under the lamp light. The micrographs were taken at an accelerated voltage of 100 kv.

### 2.5. Quantification for Raloxifene (RXF)

Raloxifene in all samples was determined by Agilent 1100 HPLC (Agilent, Santa Clara, CA, USA) coupled with an API 4000 triple quadrupole mass spectrometry (Applied Biosystems, Foster City, CA, USA). The compound of 4-(6,6-dimethyl-4-oxo-3-(trifluoromethyl)-4,5,6,7-tetrahydro-1*H*-indazol-1-yl)-2-((1r,4r)-4-hydroxycyclohexylamino)benzamide (SNX-2112) was designated as an internal standard substance for accurate quantification. The HPLC system was equipped with a degasser, a quaternary pump, an auto sampler and a VWD detector. The samples were eluted against a Kromasil C_18_ column (3.5 μm, 100 mm × 4.6 mm) at 40 °C using a gradient of formic acid (0.1%) in water (mobile phase A) vs. formic acid (0.1%) in acetonitrile (mobile phase B) at a flow rate of 0.45 mL/min and detected at 288 nm [8]. Quantitation was based on the extracted positive ion chromatograms. 

### 2.6. In Vitro Release Study

The release of RXF from RXF-ccNPs was investigated in vitro in water, 0.1 N HCl and pH 6.8 PBS. Briefly, 5 mL of RXF-ccNPs were put in into 900 mL of release medium and maintained at 37 °C under stirring (100 rpm). At predetermined intervals, 2 mL of medium were withdrawn and immediately centrifuged at 10,000 rpm using a centrifugal filter with a molecular weight cut-off (MWCO) of 10,000 (Amicon^®^ Ultra 0.5 mL, Merck, Darmstadt, Germany). RXF in the supernatants was quantified by HPLC described above. All experiments were performed in triplicate.

### 2.7. Bioavailability Study

Sprague Dawley (SD) rats (male, 220 ± 20 g) were used to appreciate the oral pharmacokinetics. The animals were treated as per the Guidelines on the Care and Use of Animals for Scientific Purposes (2004) and the experimental protocols were approved by the Institutional Review Board of Guocaoxiafang Biotechnology (GB20171203). The rats were fasted overnight but ad lib accessible to water. Rats were randomly divided into 3 groups (*n* = 6). They were administered with RXF suspensions, RXF-SICs solution and RXF-ccNPs by gavage at the dose of 50 mg/kg, respectively. Here, the suspensions formulation was prepared by dispersing RXF in 0.5% CMC-Na solution. At predetermined time points, approximately 0.25 mL of blood were collected into heparinized tubes from the tail vein of rats. The blood was immediately centrifuged at 5000 rpm for 5 min to prepare the plasma. A deproteinization procedure was utilized to extract RXF from the plasma [16]. Five aliquots of acetonitrile was added into one aliquot of plasma followed by vortex for several minutes. Then, the mixture was centrifuged for 10 min at 10,000 rpm. The supernatant was transferred to centrifuge tubes and subjected to vacuum evaporation at 37 °C using a concentrator (Concentrator Plus, Eppendorf, Hamburg, Germany). The residues were finally reconstituted in 100 μL of mobile phase for HPLC/MS analysis. Non-compartmental model was applied to process the data and extract the pharmacokinetic parameters of RXF with PKSolver 2.0, a freely available Excel program. 

### 2.8. In Situ Single-Pass Intestinal Perfusion

In situ single-pass intestinal perfusion was adopted to evaluate the intestinal permeability of free RXF and RXF-ccNPs as described by Li et al. [17]. Briefly, SD rats were fasted overnight before perfusion. Surgical procedures were performed on the rats after anesthesia with an intraperitoneal injection of 20% urethane (1.0 g/kg). A midline longitudinal incision was made to expose the abdomen. A length of 10 cm of duodenum, jejunum and ilium were cannulated with silicone tubes (Φ 2.5 × 4 mm) on both ends. The perfusion solutions were prepared in Krebs Ringer’s buffer (pH 7.4) containing 50 µg/mL of RXF or RXF-ccNPs. After pre-perfusion for 0.5 h, the effluents were collected every 15 min up to 120 min. Finally, the radius and length of various intestinal segments were measured. To minimize the error of *C*_out_, the net water flux was calibrated by weight method in comparison with the sham-operated group. The effective permeability coefficient (*P*_eff_) was calculated with the following equation:

$$P_{eff}=\frac{Q}{2\pi rL}\ln\frac{C_{out}}{C_{in}}$$

where *Q* represents the flow rate (0.2 mL/min), *r* and *L* denote the radius and length of the perfused intestinal segment (cm), *C*_in_ and *C*_out_ are the inlet and outlet concentration of RXF, respectively.

### 2.9. Cellular Uptake and Cytotoxicity

Caco-2 cells from ATCC were cultured in DMEM, in which fetal bovine serum (FBS, 10%) and penicillin-streptomycin solution (100 IU/mL) were supplemented. For cellular uptake study, Caco-2 cells were seeded in 12-well plate at a density of 5 × 10^5^ cells/well and cultured for two days under a 37 °C/5% CO_2_ atmosphere. Afterwards, the culture medium was removed and replaced with DMEM-diluted RXF or RXF-ccNPs (25 μg/mL). The cells were treated for 2 h and 4 h at 37 °C respectively, then washed twice with cold PBS, and followed by lysis with RIPA buffer. The lysates were then centrifuged at 10,000 rpm for 5 min under 4 °C to obtain the supernatant. RXF in the supernatant was determined by HPLC/MS established above. The cell concentration was gauged by protein content using a BCA protein assay kit.

The cytotoxicity of nanoparticles was evaluated by MTT assay. The well-cultured Caco-2 cells were washed thrice with PBS. Then, blank ccNPs with various levels were added into the cells and incubated for 24 h at 37 °C. After that, MTT solution (20 μL, 5 mg/mL) was added and incubated for another 4 h. At last, DMSO was used to dissolve the resultant formazan. The absorbance in the microwells was measured at 570 nm with a microplate reader (PR 4100, Bio-RAD, Hercules, CA, USA). The cell viability was calculated using the equation: Cell viability (%) = (*A*_tri_/*A*_con_) × 100%, where *A*_tri_ and *A*_con_ representing the absorbance of living cell treated with drug and blank culture medium, respectively.

### 2.10. Ex Vivo Imaging of Transepithelial Transport

The transepithelial transport characteristics of RXF-ccNPs was examined by ex vivo imaging of intestinal tissue slices. Fluorescent RXF-ccNPs were prepared through incorporating a small quantity of CY3-chitosan in the formulation of RXF-ccNPs following the same procedure described above. SD rats were fasted overnight and then orally administered with fluorescent RXF-ccNPs. After two hours, the rats were put to death, and the whole small intestine was excised immediately. In the middle of the duodenum, jejunum and ileum, a length of ca. 0.5 cm was cut off and flushed with ice-cold saline. The clean intestinal segments were fixed by 4% paraformaldehyde and then prepared into paraffin slices. The fluorescence distribution of RXF-ccNPs in the absorptive epithelia was visualized by CLSM.

## 3. Results and Discussion

### 3.1. Solubility Diagram of Raloxifene (RXF) vs. Sulfobutylether-β-Cyclodextrin (SBE-β-CD)

**Figure 1:**
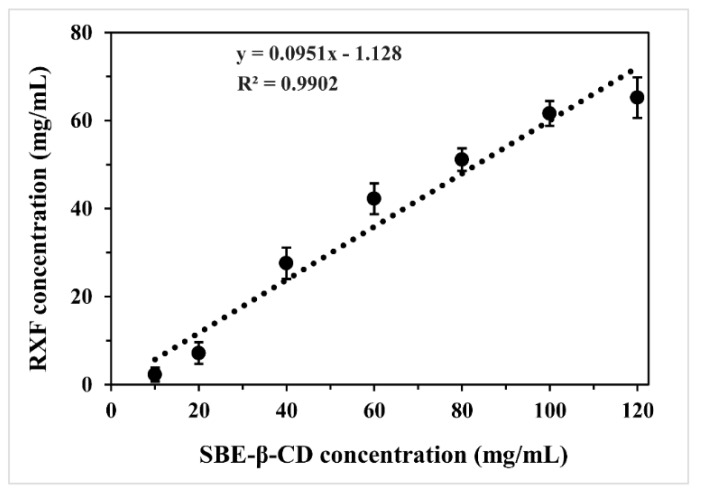
Phase solubility diagram plotted with dissolved raloxifene (RXF) vs. different concentrations of SBE-β-CD in water.

The phase solubility diagram of RXF vs. SBE-β-CD in water is shown in Figure 1. The complexation between RXF and SBE-β-CD was identified to be an A_L_-type by linear fitting (*R*^2^ = 0.9902). This type of plot indicated the formation of soluble cyclodextrin inclusion complexes at a stoichiometric rate of 1:1. The intrinsic solubility of RXF was approximately 0.465 mg/mL (9.83 × 10^−4^ mol/L) determined in our laboratory, close to that reported by Tran TH et al. [18]. Thus, the calculated complex formation constant (*K*_f_) was 106.91 L/mol. The *K*_f_ value was considerably large for an inclusion compound, demonstrating a stable complexation. Phase solubility study indicates that it is practicable to use SBE-β-CD to include RXF.

### 3.2. Preparation and Characterization of RXF-ccNPs

**Figure 2:**
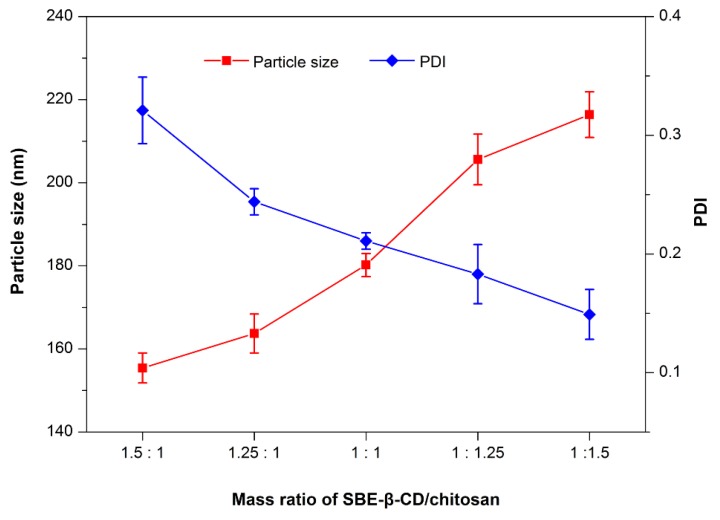
Effect of the mass ratio of sulfobutylether-β-cyclodextrin (SBE-β-CD)/chitosan in the formulation on particle size and PDI of raloxifene loaded cyclodextrin/chitosan nanoparticles (RXF-ccNPs).

The electrostatic complexation technique is a commonly used approach to fabricate polyelectrolyte nanoparticles [19,20]. In the present study, SBE-β-CD and chitosan constitute the ion counterparts that can form nanoparticles in a suitable ratio by self-assembly. The effects of mass ratio of SBE-β-CD to chitosan on particle size and PDI are illustrated in Figure 2. The particle size of RXF-ccNPs increased with the increase of SBE-β-CD/chitosan ratio, but the PDI of RXF-ccNPs moved in the opposite way. It is easily understood that high ratio of chitosan will thicken the shell of nanoparticles due to more chitosan molecules attachment, which in turn contributes to high condensation to the nanoparticles that uniforms them eventually. In general, small particle size and low dispersity are helpful for oral drug absorption. Taking the particle size and PDI together, we finally determined the mass ratio of SBE-β-CD/chitosan to be 1.25:1.

**Figure 3:**
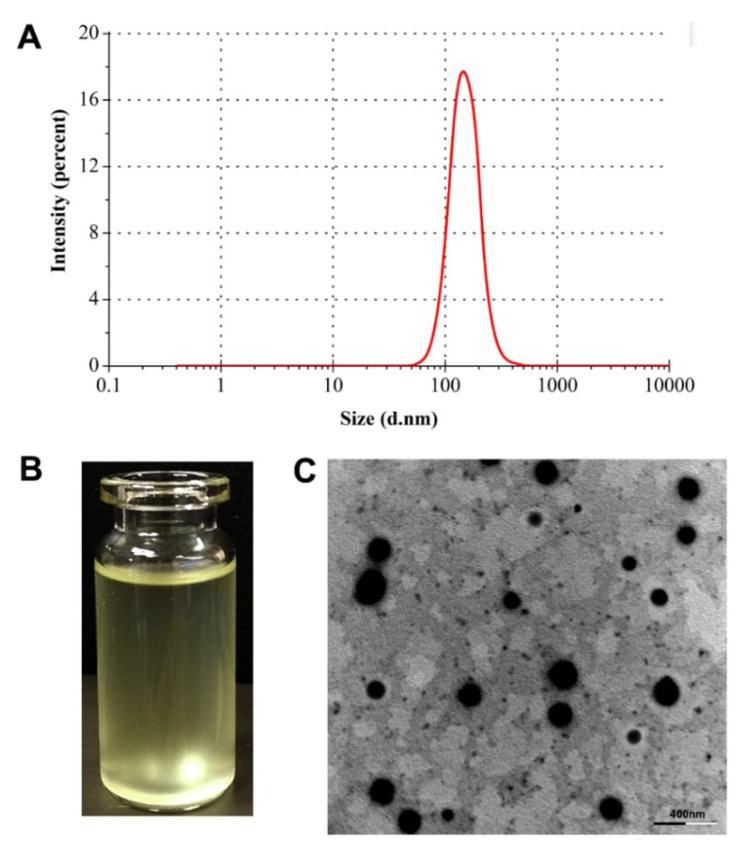
Characterization of RXF-ccNPs: particle size distribution (**A**); appearance (**B**) and TEM micrograph (**C**).

The particle size of RXF-ccNPs prepared by the formulation above established was 165.8 nm, showing a narrow distribution (PDI = 0.137) (Figure 3A). Due to poor water-solubility, most of RXF were entrapped in SBE-β-CD in the aqueous solution. After forming nanoparticles, there were over 90% RXF encapsulated into nanoparticles. The rest was solubilized RXF (free or its cyclodextrin complexes) that had less effect on the intestinal absorption. RXF-ccNPs were positively charged with a ζ potential of 26.9 mv. The resulting nanosuspensions of RXF-ccNPs appeared a transparent pale yellow (Figure 3B). RXF-ccNPs displayed a spherical morphology as observed by SEM (Figure 3C). The particle size was below 200 nm estimated by the scale bar, which was in agreement with the hydrodynamic size measured by dynamic light scattering. SEM micrograph provided direct evidence that RXF-SICs and chitosan formed nanoparticles in water by electrostatic complexation.

### 3.3. In Vitro Release of RXF-ccNPs

**Figure 4:**
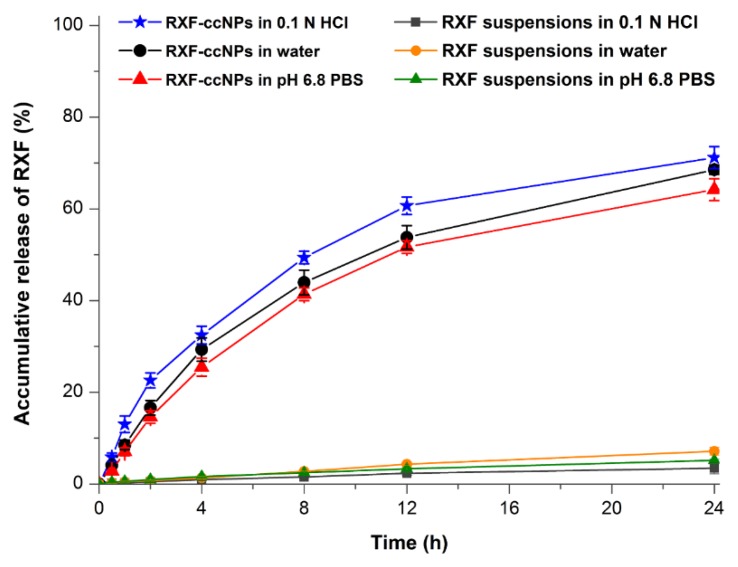
Release curves of RXF from RXF-ccNPs performed in water, 0.1 N HCl and pH 6.8 PBS based on in situ release method (*n* = 3, mean ± SD).

The in vitro release profiles of RXF from RXF-ccNPs with RXF suspensions as control are shown in Figure 4. RXF suspensions presented extremely slow drug release in all media and the accumulative release percentages were less than 10% in solubilizer-free media within 24 h. RXF-ccNPs exhibited similar drug release in three different media. However, there was no burst release effect taking place on RXF-ccNPs. On the contrary, a sustained release was provided with RXF RXF-ccNPs. It indicates that RXF can release from nanoparticles, but the release is limited due to encapsulation. The electrostatic complexation disenables the dissolution of RXF-SICs that results in a slow release. We fitted the release data using various release models [21]. The resulting correlation coefficients (*r*^2^) were 0.7862 for Zero-order, 0.9243 for First-order, 0.8351 for Higuchi model, 0.8654 for Hixson–Crowell model, and 0.8718 for Korsmeyer–Peppas model, respectively. It could be inferred that RXF release from RXF-ccNPs was in agreement with the first-order kinetic process, which could be ascribed to passive diffusion. The accumulative release was less than 33% at 4 h and approximately 70% within 24 h. In 0.1 N HCl, RXF-ccNPs presented a somewhat fast RXF release relative to in water and in pH 6.8 PBS. However, the similarity factors (*f*_2_) between three release curves were larger than 70, indicating no significant difference among them [22]. Low drug release allows RXF to be transported across the intestinal epithelia in the form of intact nanoparticles, which is favorable to break through the absorption barrier.

### 3.4. Enhanced Oral Bioavailability

**Figure 5:**
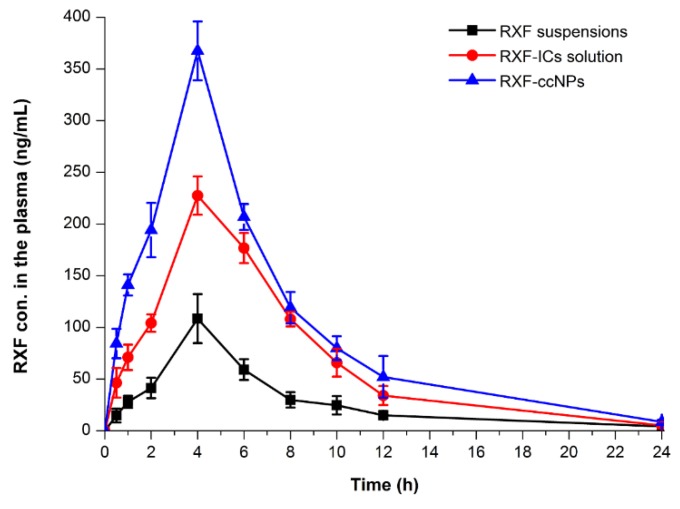
Pharmacokinetic profiles of RXF in rats after oral administration of RXF suspensions, RXF-SICs solution and RXF–ccNPs at a dose of 50 mg/kg (*n* = 6, mean ± SD).

The pharmacokinetic profiles of RXF concentration vs. time in rats after oral administration of RXF suspensions, RXF-SICs solution and RXF-ccNPs are displayed in Figure 5, and the main pharmacokinetic parameters are summarized in Table 1. For the formulation of RXF suspensions, the drug absorption was relatively inadequate with the maximal plasma concentration (*C*_max_) of 108.46 ng/mL. Different from RXF suspensions, RXF-SICs solution gave rise to an elevated *C*_max_, which was almost two times as high as that of RXF suspensions. However, RXF-ccNPs resulted in a higher *C*_max_, up to 367.48 ng/mL. The time to *C*_max_ (*t*_max_) and the terminal half-life (*t*_1/2_) among three formulations appeared to be a little distinct. The formulation with good absorption possesses smaller *t*_max_ and shorter *t*_1/2_, which is related to the fast drug disposition, including absorption and elimination. The area under plasma drug concentration vs. time curve (*AUC*_0-t_) of RXF-ccNPs was up to 2400.78 ng/mL·h that was notably larger than that of RXF suspensions and RXF-SICs solution. The oral bioavailability of RXF-ccNPs relative to RXF suspensions was calculated to be 360.76%, whereas it was just 249.38% for RXF-SICs solution. RXF-ccNPs demonstrated an excellent absorption-promoting effect on RXF.

Cyclodextrin inclusion has been shown able to promote the oral absorption of poorly water-soluble drug by improving the dissolution [23,24]. In our study, cyclodextrin inclusion complexes as a bioavailability enhancer was verified again in the case of RXF-SICs solution. However, cyclodextrin inclusion complexes are just intermediates of preparations that need to be further processed into a final dosage form. In order for ease of use, Zhang, X., et al. [24] developed a ready-to-use nanosuspension formulation containing oridonin/HP-β-cyclodextrin inclusion complexes. Dry suspensions prepared from RXF/HP-β-cyclodextrin inclusion complexes have also been attempted for formulation innovation [15]. These formulation strategies greatly carry forward the development of preparations involving cyclodextrin inclusion complexes. Our developed RXF-ccNPs is not only convenient for use, but also can enhance the oral bioavailability of RXF. 

### 3.5. Improved Intestinal Permeability

The *P*_eff_ values of free RXF and RXF-ccNPs in various intestinal segments are listed in Table 2. In the duodenum and jejunum, RXF in the form of free molecule exhibited lower permeability with *P*_eff_ smaller than 5 × 10^−6^ cm/s. It indicates that the intestinal permeability of free RXF is poor, since a *P*_eff_ of ca. 10^−6^ cm/s is predicted to be difficultly absorbed by the intestine [25]. The *P*_eff_ notably increased for ccNPs-encapsulated RXF where the *P*_eff_ values came to the order of 10^−5^ cm/s. In terms of different intestinal segments, a more significant improvement in the permeability took place in the ilium. The *P*_eff_ value of RXF-ccNPs was up to 2 × 10^−4^ cm/s, suggesting a high absorption in this site. It can be attributed to the facilitating effect from chitosan. Depending on polycationic property, chitosan-based nanoparticles can protect labile drugs against rapid degradation and interact with the enterocytes to overcome the transport barriers by opening the tight junctions and positive cytosis [26]. 

RXF available on the market is mostly the hydrochloride form. Low permeability of free RXF can be explained by the dissociation of the drug in the intestinal lumen. In addition, the determination of *P*_eff_ did not exclude the effect of intestinal metabolism on drug elimination. If the intestinal metabolism being taken into account, the *P*_eff_ of free RXF may be lower. Thus it follows that RXF-ccNPs greatly boost the intestinal permeability of RXF that is fairly favorable for RXF absorption after oral administration.

### 3.6. Excellent Cellular Uptake and Biocompatibility

**Figure 6:**
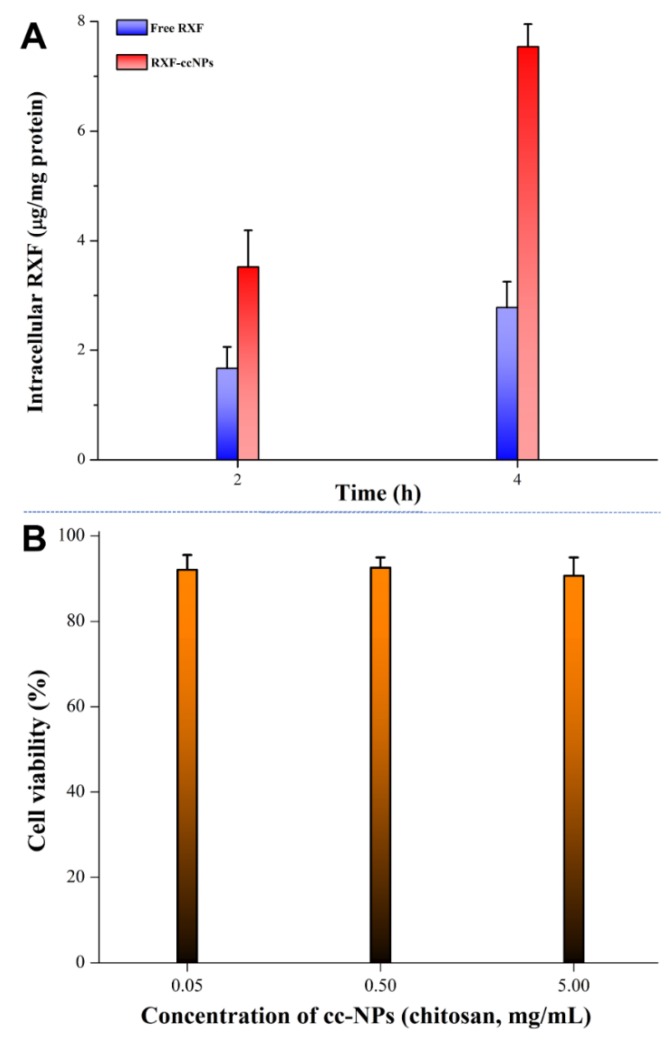
Cellular uptake quantified by intracellular RXF concentration (**A**) and cell viability of Caco-2 cells treated with ccNPs with different levels of chitosan (**B**).

High cellular uptake guarantees good absorption and bioavailability. Figure 6A shows the intracellular RXF concentration of free RXF and RXF-ccNPs after incubation with Caco-2 cells for 2 h and 4 h. It provides clear evidence that RXF-ccNPs are more easily taken up by Caco-2 cells compared with free RXF. The low bioavailability of RXF is not only associated with its poor water solubility, but also has something to do with the intestinal metabolism and efflux [27]. The ccNPs as nanocarriers can facilitate RXF influx into cells by shielding the unwanted biochemical properties. In general, molecular drug can approach to and enter into the cells more readily. In this case, the cellular uptake of RXF-ccNPs significantly exceeded that of free RXF, indicating nanoencapsulation able to overcome the cellular trafficking hindrance of RXF.

The safety of biomaterials is a great concern for oral drug delivery. Figure 6B exhibits the cytotoxicity of our developed ccNPs with low, medium and high chitosan levels. It could be seen that there was no obvious cytotoxic effect with ccNPs. The survival rates of Caco-2 cells were all larger than 90% when treated with ccNPs for 4 h. The nano-formulation just consists of SBE-β-CD and oligochitosan. These two materials have been extensively documented to be biocompatible. Accordingly, the carrier of ccNPs can be trustingly used for oral administration.

### 3.7. Good Transepithelial Capacity

**Figure 7:**
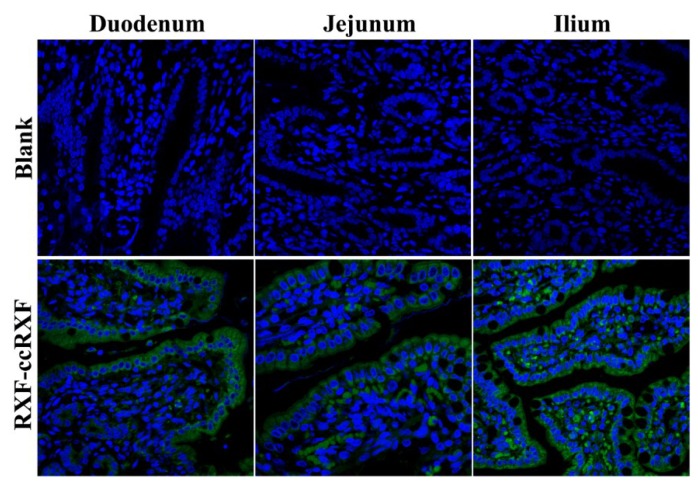
Ex vivo imaging of intestinal tissue sections after oral administration of CY3-labeled RXF-ccNPs inspected by CLSM. Green represents CY3-associated nanoparticles, and the cell nuclei were dyed with DAPI with 600X magnification.

The histomorphology of absorptive epithelia as well as fluorescence distribution on them after oral administration of fluorescent RXF-ccNPs are shown in Figure 7. In the blank groups, the epithelial villi or their longitudinal sections were clearly presented. Apparent fluorescence appeared in the fluorescent RXF-ccNPs-treated groups, showing strong intestinal affinity and permeability of RXF-ccNPs. The fluorescent infiltration was more intense for the intestinal segment of ileum. It illustrates that the ileum plays a predominant role in absorption of RXF-ccNPs. In addition, considerable fluorescence or dots were located in the central lacteals inside the villi. Generally, large colloidal particles are difficult to pass through the vascular endothelium and will preferentially transport toward the lymphatic vessel of high permeability [28]. Apart from loosening the tight junctions, chitosan-modified nanoparticles can significantly increase the lymphatic uptake amount of payload through the Peyer’s patches of the intestine [29]. High accumulation in the central lacteals renders RXF-ccNPs easier to be transported into the lymphatic system. Lymphatic transport can reduce the hepatic first-pass effect, which further contributes the enhancement of oral bioavailability.

## 4. Conclusions

We have successfully developed an oral nano-delivery system of RXF based on cyclodextrin and chitosan. It found that RXF could be well included into SBE-β-CD and further form nanoparticles with oligochitosan via electrostatic interaction. The resulting nanoparticles (cc-NPs) possessed a small particle size and exhibited a sustained drug release. The oral bioavailability of RXF was significantly enhanced through cc-NPs, up to 3.6 folds relative to RXF suspensions. RXF-ccNPs were provided with high intestinal permeability, excellent cellular uptake and biocompatibility as well as good transepithelial aptness. These findings support the assumption of ccNPs as a safe and efficient vehicle for oral delivery of RXF. The reported system provides a formulation strategy to tackle amphiphobic drugs that may serve the settlement of low bioavailability thereof.

## Figures and Tables

**Table 1:** Pharmacokinetic parameters of RXF in rats following oral administration of RXF suspensions, RXF-SICs solution and RXF-ccNPs at the dose of 50 mg/kg (*n* = 6, mean ± SD).

Parameter	RXF Suspensions	RXF-SICs Solution	RXF-ccNPs
*C*_max_ (ng/mL)	108.46 ± 12.62	227.78 ± 23.17 **	367.48 ± 26.22 **
*T*_max_ (h)	4.13 ± 0.54	3.55 ± 0.46	3.87 ± 0.51
*T*_1/2_ (h)	5.66 ± 0.78	3.58 ± 0.39 *	4.45 ± 0.43 *
*AUC*_0-t_ (ng/mL·h)	665.47 ± 39.62	1659.56 ± 74.02 **	2400.78 ± 83.26 **
*Relative BA* (%)	/	249.38	360.76

Statistical significance: ANVOA, * *p* < 0.05; ** *p* < 0.01, compared with RXF-SICs solution.

**Table 2:** The effective intestinal permeability (*P*_eff_) of free RXF and RXF-ccNPs determined by in situ single-pass intestinal perfusion (*n* = 3).

Intestinal Segment	*P*_eff_ (cm/s)	
	Free RXF	RXF-ccNPs
Duodenum	2.051 ± 0.163 × 10^−6^	6.325 ± 0.472 × 10^−5^ **
Jejunum	4.469 ± 0.278 × 10^−6^	8.224 ± 0.396 × 10^−5^ **
Ileum	1.236 ± 0.107 × 10^−5^	2.005 ± 0.514 × 10^−4^ **

Paired *t*-test, ** *p* < 0.01, compared with free RXF.
